# More women than men achieve PhDs in Swedish family medicine but scientific impact was not gender skewed - a secular trend analysis 1980–2020.

**DOI:** 10.1080/02813432.2021.1959713

**Published:** 2021-08-04

**Authors:** Ferdinando Petrazzuoli, Hans Thulesius

**Affiliations:** Post doc researcher, Lund University, Department of Clinical Sciences, Division of Family Medicine, Malmö, Sweden; Department of Research and Development, Region Kronoberg, Växjö, Sweden; Lund University, Department of Clinical Sciences, Division of Family Medicine, Malmö, Sweden, Linnaeus University, Department of Medicine and Optometry, Kalmar, Sweden

To achieve a PhD in primary care or family medicine has been similar in the five Nordic countries: Denmark, Finland, Iceland, Norway and Sweden; yet, there were variations in procedures [1]. In the following, we will analyze the scientific impact of the secular trend of a higher woman to man ratio of the physician PhD researchers using all 296 PhDs associated with primary care university departments in Sweden 1980–2020 as an example. This secular trend of more women than men PhDs is not unique for family medicine, and a study of Swedish biomedical doctoral dissertations 1991–2009 reported it as well [2]. Also, women achieved 71% of doctoral degrees in health and medical sciences in the United States 2018–2019 [3].

A secular trend is that component of a time series that gives the data’s general tendency for an extended period [4]. A secular trend can be seen in many western countries regarding the increasing proportion of women to men graduate and doctoral students [3].

We, FP (2019) and HT (2003), are men authors of 2 out of 296 PhDs by physicians at seven Swedish university departments of primary care 1980–2020. While HT belonged to a majority gender group of PhD candidates in 2003, FP belonged to a minority gender group in 2019 [5].

For 258 physicians, the PhDs were defended at primary care departments and 38 at other departments; 44% of the PhDs came from other departments between 1980 and 1990 and 1% between 2011 and 2020. According to administrators and professors at the seven universities, the list of dissertations is complete, but we have not confirmed it by the use of external sources of information.

**Table 1.** PhD primary care physician dissertations at Swedish universities 1980–2020.

	1980–1990	1991–2000	2001–2010	2011–2020	All years 1980–2020
Men, PhD	25 (69%)	49 (72%)	58 (59%)	36 (39%)	168 (57%)
Women, PhD	11 (31%)	19 (28%)	41 (41%)	57 (61%)	128 (43%)
Total	36 (100%)	68 (100%)	99 (100%)	93 (100%)	296 (100%)
Dissertation of the year award, Men			6 (60%)	2 (20%)	8 (40%)
Dissertation of the year award, Women			4 (40%)	8 (80%)	12 (60%)
*Scandinavian Journal of Primary Health Care* publications, median number (range), Men	4 (0–24	2 (0–24)	1 (0–17)	0 (0–8)	1 (0–24)
*Scandinavian Journal of Primary Health Care* publications, median number (range), Women	1 (0–31)	2 (0–6)	1 (0–11)	1 (0–5)	1 (0–31)
Mann–Whitney *U*-test for Men vs Women difference	*p* = 0.25	*p* = 0.14	*p* = 0.98	*p* = 0.14	*p* = 0.053
Google Scholar citation numbers, crude/adjusted, Men	266/46^a^	185/74^a^	133/85^a^	54/46^a^	151/68^a^
Google Scholar citations numbers, crude/adjusted^a^, Women	138/25^a^	125/59^a^	110/70^a^	44/39^a^	85/51^a^
Mann–Whitneys *U*-test, crude/adjusted^a^ for Men vs Women difference	*p* = 0.48/0.31^a^	*p* = 0.22/0.52^a^	*p* = 0.99/0.95^a^	*p* = 0.20/0.25^a^	*p* = **0.01**/0.41^a^

^a^Adjustment algorithm: I. Year order factor (YoF) according to 1980–1981 = 1, 1982–1983 = 2, 1984–1985 = 3….2018–2019 = 20, 2020 = 21. II: YoF multiplied by crude number of Google Scholar citations for most cited publication divided by 21. p-value in bold shows significance.

We analyzed the gender distribution of PhD candidates during the four decades since the first three primary care physicians defended their theses in 1980.

Between 1980 and 1990 a clear majority of the PhD candidates were men, while this distribution flipped so that between 2011 and 2020 a clear majority of the PhD candidates were women (see Table 1).

We further compared the gender proportion of the 20 PhD candidates given the dissertation of the year award by the Swedish Association of General Practice (SFAM) 2001–2020.

The same distribution as in the number of PhD candidates was seen with more men receiving the award between 2001 and 2010 and more women receiving the award between 2011 and 2020 (see Table 1). In 2020 a woman researcher was again given the award, and her dissertation about patient safety received Swedish media attention [6,7].

To get one measure of how much impact the candidates’ research has had on the Nordic primary care scientific discourse, we compared the total number of publications in the *Scandinavian Journal of Primary Health Care* by the 296 researchers.

As in the previous comparisons, the dominance of publications by men researchers in 1980–1990 was changed into a higher median number of publications by women researchers 2011–2020, yet both differences were non-significant (see Table 1).

Finally, to compare the scientific contribution made by the researchers in terms of the number of citations we noted and compared the single most cited publication according to Google Scholar of each PhD candidate 1980–2020. We developed an algorithm and calculated an adjusted number of citations according to the year order of the dissertation to adapt to the fact that the age of a publication correlates to how much it gets cited. As in the comparison of the number of *Scandinavian Journal of Primary Health Care* citations, the men researchers dominated in the number of Google Scholar citations 1980–1990 while this difference was levelled out in 2011–2020 and the differences were not significant apart from the crude comparison over the entire period 1980–2020. When compensating for the fact that older publications give more citations using an adjustment algorithm, this gender difference was not significant.

To conclude, the secular trend of more women than men achieving PhDs in the western world [2,3] was confirmed in this study of all 296 PhDs by physicians associated with primary care university departments in Sweden 1980–2020. Yet, no skewed relationship based on gender was seen when we compared the scientific impact of the research achievements by three different methods: awarded dissertations, the number of publications in the *Scandinavian Journal of Primary Health Care* and number of Google Scholar citations of single best publication when compensating for the age of the publication. This lack of skewed gender difference in scientific performance was also seen in a longitudinal study of health science researchers [8].

The trend of more women than men PhDs in primary care aligns with the ‘feminisation’ of the primary care physician workforce as seen in a systematic review [9]. However, the clinical primary care leadership [10], as well as the senior academic leadership in primary care, is still dominated by men [11]. Let us hope that this only reflects inertia caused by the fact that it takes time to become a professor.
